# Supplementary material for: Large vesicle extrusions from C. elegans neurons are consumed and stimulated by glial-like phagocytosis activity of the neighboring cell
Source: eLife. 2023 Mar 2;12:e82227. doi: 10.7554/eLife.82227 (PMC10023159; doi:10.7554/eLife.82227)
Supplement: Figure 1—source data 1. [file elife-82227-fig1-data1.docx]

**Numerical data for Figure 1B –** Time from exopher identification to first observation of exopher vesiculation is graphed.

| Sample | Day1 sn onset,hour | Day2 sn onset,hour |
| --- | --- | --- |
| 1 | 0.87 | 5.22 |
| 2 | 0.65 | 3.68 |
| 3 | 0.55 | 7.3 |
| 4 | 0.5 | 5.13 |
| 5 | 0.45 | 3.57 |
| 6 | 0.63 | 3.5 |
| 7 | 2 | 3.32 |
| 8 | 1.77 | 1.25 |
| 9 | 1.23 | 1.17 |
| 10 | 1.42 | 1.13 |
| 11 | 20.28 | 1.07 |
| 12 | 2.333333333 | 0.666666667 |
| 13 | 0.766666667 | 0.516666667 |
| 14 | 0.75 | 0.15 |
| 15 | 0.583333333 | 0.833333333 |
| 16 | 2.85 | 0.583333333 |
| 17 | 1 | 1.683333333 |
| 18 | 2.616666667 | 1.483333333 |
| 19 | 0.766666667 | 0.566666667 |
| 20 | 2.233333333 | 0.883333333 |
| 21 | 1.65 | 5.633333333 |
| 22 | 0.533333333 | 5.45 |
| 23 | 0.45 |  |
| 24 | 1.05 |  |
| 25 | 2.866666667 |  |
| 26 | 2.533333333 |  |
| 27 | 2.45 |  |
| 28 | 1.583333333 |  |
| 29 | 0.4 |  |
| 30 | 3.516666667 |  |
| 31 | 1.583333333 |  |
| 32 | 2.333333333 |  |
|  |  |  |
| mean | 2.04 | 2.49 |
|  |  |  |
| Comparison | P-Value |  |
| Day1 vs day2 | 0.58577 |  |

**Numerical data for Figure 1C –** Time from the start of exopher vesiculation to the observed loss of exopher-derived mCherry signal is graphed.

| sample | Day1 sn persistence,hour | Day2 sn persistence,hour |
| --- | --- | --- |
| 1 | 38.57 |  |
| 2 | 94.43 | 75.57 |
| 3 | 95.7 | 91.41666667 |
| 4 | 119.05 | 145.78 |
| 5 | 119.55 | 102.85 |
| 6 | 68.67 | 102.75 |
| 7 | 94.45 | 75.43 |
| 8 | 139.63 | 99.15 |
| 9 | 71 | 47.33333333 |
| 10 | 89 | 146.58 |
| 11 | 68.56666667 | 71.96666667 |
| 12 | 115.5833333 | 96.37 |
| 13 | 70.7 | 96.33 |
| 14 | 46.6 | 143.67 |
| 15 | 70.53333333 | 70.03 |
| 16 | 45.41666667 |  |
| 17 | 65 |  |
| 18 | 67.38333333 |  |
| 19 | 67.35 |  |
| 20 | 68.16666667 |  |
| 21 | 45.23333333 |  |
| 22 | 89 |  |
| 23 | 68.83333333 |  |
| 24 | 89 |  |
|  |  |  |
| mean | 79.48 | 97.52 |
|  |  |  |
| Comparison | P-Value |  |
| Day1 vs day2 | 0.0589 |  |
