# Supplementary material for: Large vesicle extrusions from C. elegans neurons are consumed and stimulated by glial-like phagocytosis activity of the neighboring cell
Source: eLife. 2023 Mar 2;12:e82227. doi: 10.7554/eLife.82227 (PMC10023159; doi:10.7554/eLife.82227)
Supplement: Figure 2—source data 1. [file elife-82227-fig2-data1.docx]

**Numerical data for Figure 2B –** the volume of overlapping signal in 3-D projections between the hypodermal F-ACTIN and ALMR-neuron derived exopher, comparing to the ALMR neuronal soma.

| sample |  | soma | exopher |
| --- | --- | --- | --- |
| 1 |  | 0.091773 | 4.463058 |
| 2 |  | 0.024151 | 1.159236 |
| 3 |  | 0.729353 | 1.178556 |
| 4 |  | 0.569958 | 0.089358 |
| 5 |  | 0.560297 | 0.224602 |
| 6 |  | 0.652883 | 0.15215 |
| 7 |  | 0.646781 | 0.140074 |
| 8 |  | 0 | 7.754804 |
| 9 |  | 0 | 7.718578 |
| 10 |  | 0.219772 | 10.12884 |
| 11 |  | 0.287394 | 9.652909 |
| 12 |  | 0.618259 | 1.202037 |
| 13 |  | 0.611014 | 0.793223 |
| 14 |  | 0.108678 | 0.89085 |
| 15 |  | 0.031396 | 1.712288 |
| 16 |  | 0.449204 | 1.60844 |
| 17 |  |  | 0.519241 |
| 18 |  |  | 0.246338 |
| 19 |  |  | 0.289809 |
| 20 |  |  | 0.287394 |
| 21 |  |  | 0.32362 |
| 22 |  |  | 0.845276 |
| 23 |  |  | 2.651752 |
| 24 |  |  | 2.861863 |
|  |  |  |  |
| mean |  | 0.3501 | 2.371 |
|  |  |  |  |
| Comparison |  | P-Value |  |
| Soma vs Exopher |  | 0.0150 |  |
|  |  |  |  |

**Numerical data for Figure 2D –** the volume of overlapping signal in 3-D projections between the hypodermal PI(4,5)P_2_ and ALMR-neuron derived exopher, comparing to the ALMR neuronal soma.

| sample | soma | exopher |
| --- | --- | --- |
| 1 | 0.746258023 | 0.697956533 |
| 2 | 0.338110431 | 0.152149694 |
| 3 | 0.077282384 | 2.80148643 |
| 4 | 0.1231688 | 6.112553582 |
| 5 | 0.012075373 | 5.822744641 |
| 6 | 0.021735671 | 1.458705003 |
| 7 | 0 | 0.58927818 |
| 8 | 0.234262227 | 2.047983183 |
| 9 | 0.033811043 | 2.690393003 |
| 10 | 0.272903419 | 0.316374761 |
| 11 | 0.125583874 | 0.355015953 |
| 12 | 0 | 0.456449082 |
| 13 | 0 | 0.272903419 |
| 14 | 0 | 0.707616831 |
| 15 |  | 3.431820877 |
| mean | 0.1418 | 1.861 |
|  |  |  |
| Comparison | P-Value |  |
| Soma vs Exopher | 0.0031 |  |

**Numerical data for Figure 2F** **–**exopher frequency in the hypodermal-specific RNAi for empty vector control (L4440), *act-1*, *act-2* or *act-3*

| trial | L4440 | *act-1* | *act-2* | *act-3* |
| --- | --- | --- | --- | --- |
| 1 | 4.2 | 9.2 | 13.5 | 14.6 |
| 2 | 7 | 12.3 | 21.3 |  |
| 3 | 5.6 | 10 |  |  |
| 4 | 4.3 | 10.2 | 31.1 | 14.3 |
| 5 | 5.7 | 14.3 | 26.3 | 24.2 |
| 6 | 5.4 |  | 24.5 | 33.3 |
|  |  |  |  |  |
| P-Value  Compared to L4440 |  | 0.02825 | 1.76824E-08 | 1.62555E-06 |

**Numerical data for Figure 2G** **–** exopher and starry night frequency in the hypodermal-specific RNAi for empty vector control (L4440) and *arx-2*

| trial | L4440_exopher | *arx-2_exopher* | L4440_starry night | *arx-2_starry night* |
| --- | --- | --- | --- | --- |
| 1 | 2.4 | 24.1 | 23.8 | 3.7 |
| 2 | 9.6 | 18.9 | 28.9 | 7.6 |
| 3 | 4.2 | 12 | 7.4 | 6 |
| 4 | 15.6 | 12.5 | 21.9 | 1.6 |
| 5 | 9.6 | 6 | 28.8 | 8 |
| 6 | 4 | 14.3 | 34 | 11.9 |
|  |  |  |  |  |
| P-Value  Compared to L4440 |  | 0.016652915 |  | 3.10482E-09 |
