# Supplementary material for: Large vesicle extrusions from C. elegans neurons are consumed and stimulated by glial-like phagocytosis activity of the neighboring cell
Source: eLife. 2023 Mar 2;12:e82227. doi: 10.7554/eLife.82227 (PMC10023159; doi:10.7554/eLife.82227)
Supplement: Figure 3—source data 1. [file elife-82227-fig3-data1.docx]

**Numerical data for Figure 3B –** the volume of overlapping signal in 3-D projections between the hypodermal RAB-5 and ALMR-neuron derived exopher, comparing to the ALMR neuronal soma.

| sample | soma | exopher |
| --- | --- | --- |
| 1 | 0.671391 | 0.717277 |
| 2 | 0.683466 | 0.106263 |
| 3 | 0.311545 | 0.999841 |
| 4 | 0 | 1.267914 |
| 5 | 0.007245 | 0.026566 |
| 6 | 0.019321 | 0.154565 |
| 7 | 0 | 0.036226 |
| 8 | 0.454034 | 0.801805 |
| 9 | 0.15698 | 0.077282 |
| 10 | 0.185961 | 0.062792 |
| 11 | 0.111093 | 0.299469 |
| 12 | 0 | 0.340526 |
| 13 | 0.094188 | 1.156821 |
| 14 | 0.263243 | 0.135244 |
| 15 | 0.193206 | 0.953954 |
| 16 | 0.381582 | 0.717277 |
| 17 | 0.002415 | 1.287235 |
| 18 | 0.671391 | 1.267914 |
| 19 | 1.342781 |  |
| 20 | 0.311545 |  |
|  |  |  |
| mean | 0.2931 | 0.5783 |
|  |  |  |
| Comparison | P-Value |  |
| Soma vs Exopher | 0.0419 |  |
|  |  |  |

**Numerical data for Figure 3D –** the volume of overlapping signal in 3-D projections between the hypodermal RAB-7 and ALMR-neuron derived exopher, comparing to the ALMR neuronal soma.

| sample | soma | exopher |
| --- | --- | --- |
| 1 | 0 | 0.079697 |
| 2 | 0.654485 | 1.205122 |
| 3 | 0.033811 | 0.403317 |
| 4 | 0.280679 | 1.081953 |
| 5 | 0.193206 | 2.098989 |
| 6 | 0.202866 | 0.533731 |
| 7 | 0.434713 | 6.984395 |
| 8 | 0 | 0.193206 |
| 9 | 0 | 0.217357 |
| 10 | 0 | 0.937049 |
| 11 | 0.33124 | 2.869109 |
| 12 |  | 1.787155 |
|  |  |  |
| mean | 0.1937 | 1.533 |
|  |  |  |
| Comparison | P-Value |  |
| Soma vs Exopher | 0.0320 |  |
|  |  |  |

**Numerical data for Figure 3F –** the volume of overlapping signal in 3-D projections between the hypodermal LGG-1 and ALMR-neuron derived exopher, comparing to the ALMR neuronal soma.

| sample | soma | exopher |
| --- | --- | --- |
| 1 | 0.66173 | 0.132829 |
| 2 | 0.451619 | 0.200451 |
| 3 | 0 | 0.280149 |
| 4 | 0.024151 | 0.432298 |
| 5 | 0.00966 | 0.726937 |
| 6 | 0.207696 | 0.748673 |
| 7 | 0 | 1.386253 |
| 8 | 0.077282 | 1.195462 |
| 9 | 0.057962 | 0.106263 |
| 10 | 0.036226 | 0.164225 |
| 11 | 0.15215 | 0.127999 |
| 12 | 0 | 0.002415 |
| 13 |  | 0.734183 |
|  |  |  |
| mean | 0.1399 | 0.4799 |
|  |  |  |
| Comparison | P-Value |  |
| Soma vs Exopher | 0.0242 |  |
|  |  |  |

**Numerical data for Figure 3H –** the volume of overlapping signal in 3-D projections between the hypodermal LMP-1 and ALMR-neuron derived exopher, comparing to the ALMR neuronal soma.

| sample | soma | exopher |
| --- | --- | --- |
| 1 | 0.700372 | 0.321205 |
| 2 | 1.333121 | 1.391083 |
| 3 | 4.552415 | 1.705043 |
| 4 | 1.014331 | 0.531316 |
| 5 | 0.64241 | 0.299469 |
| 6 | 0.992596 | 4.233626 |
| 7 | 2.707299 | 0.30913 |
| 8 | 2.229114 | 1.661571 |
| 9 | 0.867012 | 0.16181 |
| 10 | 0.782484 | 0.033811 |
| 11 | 3.583971 | 2.910165 |
| 12 | 3.349708 | 4.762527 |
| 13 | 0.441959 | 0.835616 |
| 14 | 1.120595 | 1.251009 |
| 15 | 0.062792 | 1.934475 |
| 16 | 0.678636 | 0.135244 |
| 17 | 0.057962 |  |
| 18 | 0.038641 |  |
| 19 | 0.598938 |  |
| 20 | 0.205281 |  |
| 21 | 2.161492 |  |
|  |  |  |
| mean | 1.339 | 1.405 |
|  |  |  |
| Comparison | P-Value |  |
| Soma vs Exopher | 0.8846 |  |
|  |  |  |
