# Supplementary material for: Large vesicle extrusions from C. elegans neurons are consumed and stimulated by glial-like phagocytosis activity of the neighboring cell
Source: eLife. 2023 Mar 2;12:e82227. doi: 10.7554/eLife.82227 (PMC10023159; doi:10.7554/eLife.82227)
Supplement: Figure 4—source data 1. [file elife-82227-fig4-data1.docx]

| sample | AMAN-2 | RME-1 | 2XFYVE | RAB-10 | RAB-7 | LMP-1 | LGG-1 |
| --- | --- | --- | --- | --- | --- | --- | --- |
| 1 | 7.968059803 | 8.93877551 | 34.22148 | 47.66469 | 74.69353 | 12.65548 | 94.13636 |
| 2 | 3.847386983 | 6.830601093 | 31.1529 | 57.08556 | 80.68006 | 20.59859 | 94.68996 |
| 3 | 4.817649707 | 3.6875 | 37.13188 | 59.49576 | 82.42131 | 11.65815 | 92.90743 |
| 4 | 5.435066632 | 2.360322677 | 87.60951 | 70.04539 | 68.97799 | 21.79834 | 58.17091 |
| 5 | 7.396528705 | 7.195571956 | 31.25846 | 74.55185 | 77.86759 | 18.89697 | 62.85842 |
| 6 | 5.486100897 | 10.01371742 | 85.90604 | 86.55914 | 85.75515 | 34.20325 | 63.80732 |
| 7 | 7.093105899 | 20.84645305 | 51.35823 | 78.23691 | 56.28295 | 33.38969 | 93.36735 |
| 8 | 4.554804584 | 17.1613904 | 70.58824 | 66.84046 | 63.18776 | 17.45236 | 92.85363 |
| 9 | 4.951488792 | 14.72287784 | 55.34466 | 74.78207 | 59.22979 | 17.6071 | 93.30752 |
| 10 | 1.529289787 | 22.541841 | 67.92793 | 67.95201 | 82.17282 | 10.95335 | 99.12034 |
| 11 | 11.3574287 | 26.00776351 | 25.45799 | 60.59682 | 88.03047 | 11.38277 | 99.15133 |
| 12 | 2.400519031 | 21.57996146 | 35.83046 | 63.16754 | 89.2517 | 9.154056 | 98.85727 |
| 13 | 2.968392121 | 14.5155113 | 46.16534 | 65.85133 | 72.58772 | 9.248555 | 96.94107 |
| 14 | 2.879377432 | 27.49904251 | 27.07424 | 64.07992 | 74.65347 | 36.17316 | 95.79419 |
| 15 | 2.199959928 | 28.89635565 | 46.62577 | 66.27396 | 73.87455 | 32.12951 | 93.66638 |
| 16 | 0.824468085 | 21.88585608 | 43.64896 | 74.32911 | 59.91858 | 14.04587 | 69.26471 |
| 17 | 1.36726468 | 29.35970849 | 40.74074 | 78.44914 | 61.0963 | 14.9553 | 70.36288 |
| 18 | 3.543934165 | 23.51123017 | 38.34259 | 73.66441 | 56.2466 |  | 67.11201 |
| 19 | 7.987220447 | 17.17620482 | 37.88648 | 78.34315 | 56.48014 |  | 56.18542 |
| 20 | 3.170671291 | 14.00791967 | 87.55531 | 72.06066 | 60.399 |  | 57.11544 |
| 21 | 5.384900074 | 14.08151439 | 85.71429 | 71.98251 | 61.49875 |  | 55.78269 |
| 22 | 5.537459283 |  | 73.40426 | 66.29605 |  |  | 92.90292 |
| 23 | 0.484166667 |  | 79.48316 | 76.76811 |  |  | 91.9836 |
| 24 | 6.635802469 |  | 62.57521 | 74.4868 |  |  | 92.85952 |
| 25 | 4.657873042 |  | 85.15008 | 71.49969 |  |  | 85.26897 |
| 26 |  |  | 70.5848 | 95.31697 |  |  | 84.59042 |
| 27 |  |  | 90.4698 | 94.31327 |  |  | 85.68475 |
| 28 |  |  | 59.73742 | 93.41062 |  |  | 75.0764 |
| 29 |  |  | 53.91517 | 84.80114 |  |  | 77.1855 |
| 30 |  |  | 50.7431 | 90.85258 |  |  | 76.25014 |
| 31 |  |  | 57.42198 | 94.86911 |  |  | 80.76543 |
| 32 |  |  | 80.99052 | 88.92734 |  |  | 82.3898 |
| 33 |  |  | 48.26139 | 84.68824 |  |  | 80.06053 |
| 34 |  |  | 83.72826 | 63.30887 |  |  |  |
| 35 |  |  | 52.87222 | 67.22831 |  |  |  |
| 36 |  |  | 51.37174 | 57.63158 |  |  |  |
| 37 |  |  | 49.62043 | 58.1854 |  |  |  |
| 38 |  |  | 47.94908 | 53.26733 |  |  |  |
| 39 |  |  | 53.13901 | 58.72576 |  |  |  |
| 40 |  |  |  | 94.53718 |  |  |  |
| 41 |  |  |  | 93.09021 |  |  |  |
| 42 |  |  |  | 95.03546 |  |  |  |
| 43 |  |  |  | 87.8453 |  |  |  |
| 44 |  |  |  | 89.60245 |  |  |  |
| 45 |  |  |  | 91.34438 |  |  |  |
|  |  |  |  |  |  |  |  |

**Numerical data for Figure 4B –** the overlap of each hypodermal marker with Starry Night in wild type
