# Supplementary material for: Large vesicle extrusions from C. elegans neurons are consumed and stimulated by glial-like phagocytosis activity of the neighboring cell
Source: eLife. 2023 Mar 2;12:e82227. doi: 10.7554/eLife.82227 (PMC10023159; doi:10.7554/eLife.82227)
Supplement: Figure 5—source data 1. [file elife-82227-fig5-data1.docx]

**Numerical data for Figure 5B –** the overlap of hypodermal LMP-1 with Starry Night in wild- type and *cup-5(ar465)* mutant

| sample | wild-type | *cup-5(ar465)* |
| --- | --- | --- |
| 1 | 12.65547878 | 75.87491 |
| 2 | 20.59859155 | 77.69617 |
| 3 | 11.65815142 | 87.09922 |
| 4 | 21.79833971 | 84.84544 |
| 5 | 18.89696855 | 75.58174 |
| 6 | 34.20325453 | 55.14277 |
| 7 | 33.38968724 | 75.5045 |
| 8 | 17.45235707 | 54.25154 |
| 9 | 17.60709568 | 72.29489 |
| 10 | 10.95334686 | 87.8392 |
| 11 | 11.38277202 | 82.25256 |
| 12 | 9.154056065 | 77.28469 |
| 13 | 9.248554913 | 55.7978 |
| 14 | 36.17316017 | 52.79715 |
| 15 | 32.12951432 | 77.65217 |
| 16 | 14.04587373 | 78.83273 |
| 17 | 14.955303210 | 96.44105 |
| 18 |  | 54.60781 |
| 19 |  | 53.67385 |
| 20 |  | 96.52439 |
|  |  |  |
|  |  |  |
|  |  |  |
|  |  |  |
|  |  |  |
| mean | 19.19 | 73.60 |
|  |  |  |
| Comparison | P-Value |  |
| WT vs mutant | 2.49522E-15 |  |
|  |  |  |

**Numerical data for Figure 5C –**the average area of Starry Night in wild-type and *cup-5(ar465)* mutant

| sample | wild-type | *cup-5(ar465)* |
| --- | --- | --- |
| 1 | 3.65547878 | 64.87491 |
| 2 | 0 | 7.83273 |
| 3 | 2.65815142 | 5.44105 |
| 4 | 3.79833971 | 53.84544 |
| 5 | 0 | 40.58174 |
| 6 | 5.20325453 | 5.79715 |
| 7 | 5.38968724 | 30.5045 |
| 8 | 1.45235707 | 24.25154 |
| 9 | 0 | 20.3 |
| 10 | 2.95334686 | 19.8392 |
| 11 | 1.38277202 | 18.25256 |
| 12 | 1.154056065 | 16.28469 |
| 13 | 3.248554913 | 8.7978 |
| 14 | 3.17316017 | 39.14277 |
| 15 | 0 | 8.65217 |
| 16 | 0 | 58.69617 |
| 17 | 2.95530321 | 55.09922 |
| 18 | 0 | 3.60781 |
| 19 | 0 | 1.67385 |
| 20 | 0 | 2 |
|  |  |  |
|  |  |  |
|  |  |  |
|  |  |  |
|  |  |  |
| mean | 1.851 | 24.27 |
|  |  |  |
| Comparison | P-Value |  |
| WT vs mutant | 2.42403E-05 |  |
|  |  |  |

**Numerical data for Figure 5D –**the average fluorescence intensity of Starry Night in wild-type and *cup-5(ar465)* mutant

| sample | wild-type | *cup-5(ar465)* |
| --- | --- | --- |
| 1 | 1200 | 2900 |
| 2 | 1230 | 2750 |
| 3 | 1240 | 2630 |
| 4 | 1330 | 2700 |
| 5 | 1400 | 2540 |
| 6 | 2000 | 2400 |
| 7 | 1800 | 1800 |
| 8 | 1890 | 1700 |
| 9 | 1500 | 1500 |
| 10 | 1300 | 1550 |
| 11 | 1425 | 1490 |
| 12 | 1330 | 1300 |
| 13 | 465 | 1200 |
| 14 | 500 | 1000 |
| 15 | 800 | 800 |
| 16 | 900 | 450 |
| 17 | 1000 | 600 |
| 18 | 650 | 1760 |
| 19 | 800 | 2240 |
| 20 | 850 | 960 |
|  |  |  |
|  |  |  |
|  |  |  |
|  |  |  |
|  |  |  |
| mean | 1181 | 1714 |
|  |  |  |
| Comparison | P-Value |  |
| WT vs mutant | 0.0099 |  |
|  |  |  |

**Numerical data for Figure 5E** **–** exopher and starry night numbers in wild-type and *sand-1(or552)* mutant

| trial | wild-type exopher | *sand-1(or552) exopher* | wild-type starry night | *sand-1(or552) starry night* |
| --- | --- | --- | --- | --- |
| 1 | 8.3 | 24 | 9.6 | 1.8 |
| 2 | 6 | 26 | 6 | 2.2 |
| 3 | 3.9 | 30 | 8.6 | 1.5 |
|  |  |  |  |  |
| P-Value  Compared to L4440 |  | 3.77186E-06 |  | 0.028116304 |

**Numerical data for Figure 5F** **–** exopher numbers at different days of adulthood in wild-type and *sand-1(or552)* mutant

| day | wild-type | | | *sand-1(or552)* | | |
| --- | --- | --- | --- | --- | --- | --- |
| 1 | 3.7 | 2.9 | 0 | 18.9 | 24 | 26 |
| 2 | 11.1 | 6 | 7.4 | 27.5 | 31.6 | 32 |
| 3 | 4 | 6 | 8.6 | 35.7 | 37 | 28 |
| 4 | 1.9 | ND | ND | 21.7 | ND | ND |
