# Supplementary material for: Large vesicle extrusions from C. elegans neurons are consumed and stimulated by glial-like phagocytosis activity of the neighboring cell
Source: eLife. 2023 Mar 2;12:e82227. doi: 10.7554/eLife.82227 (PMC10023159; doi:10.7554/eLife.82227)
Supplement: Figure 5—figure supplement 1—source data 1. [file elife-82227-fig5-figsupp1-data1.docx]

**Numerical data for Figure 5 - Figure supplement 1A –** frequency ratio of animals displaying Day 3 Starry Night vesicles to those positive for intact exopher-phagosomes on Day 2, comparing wild-type and *arl-8(wy271)* mutant.

| trial | wild-type | *arl-8(wy271)* |
| --- | --- | --- |
| 1 | 0.833 | 0.2 |
| 2 | 0.65 | 0.26 |
| 3 | 0.68 | 0.3 |
|  |  |  |
| P-Value  Compared to wild-type |  | 0.0018 |

**Numerical data for Figure 5 - Figure supplement 1B** **–** exopher numbers at different days of adulthood in wild-type and *arl-8(wy271)* mutant

| day | wild-type | | | *arl-8(wy271)* | | |
| --- | --- | --- | --- | --- | --- | --- |
| 1 | 3.7 | 0 | 2.3 | 3.6 | 2.8 | 1.9 |
| 2 | 11.1 | 6 | 7.2 | 7.5 | 9 | 6.8 |
| 3 | 4 | 6 | 8.6 | 25.5 | 21 | 19 |
| 4 | 1.9 | 2 | 3 | 16.7 |  | 14 |

**Numerical data for Figure 5 - Figure supplement 1B** **–** starry night numbers at different days of adulthood in wild-type and *arl-8(wy271)* mutant

| day | wild-type | | | *arl-8(wy271)* | | |
| --- | --- | --- | --- | --- | --- | --- |
| 1 | 0 | 0 |  | 0 | 0 |  |
| 2 | 6.8 | 6 | 7.9 | 0 | 0 | 0 |
| 3 | 4 | 6 | 8.6 | 2.1 | 4 | 2.8 |
| 4 | 7.5 |  |  | 12.5 | 9 |  |
