# Supplementary material for: Large vesicle extrusions from C. elegans neurons are consumed and stimulated by glial-like phagocytosis activity of the neighboring cell
Source: eLife. 2023 Mar 2;12:e82227. doi: 10.7554/eLife.82227 (PMC10023159; doi:10.7554/eLife.82227)
Supplement: Figure 6—source data 1. [file elife-82227-fig6-data1.docx]

**Numerical data for Figure 6A –** the volume of overlapping signal in 3-D projections between the hypodermal CNT-1 and ALMR-neuron derived exopher, comparing to the ALMR neuronal soma.

| sample | soma | exopher |
| --- | --- | --- |
| 1 | 0 | 0.277734 |
| 2 | 0 | 0.412978 |
| 3 | 0 | 0.207696 |
| 4 | 0 | 0.248753 |
| 5 | 0.644825 | 2.354698 |
| 6 | 0.130414 | 1.371762 |
| 7 | 0.00483 | 0.036226 |
| 8 | 0.026566 | 0.560297 |
| 9 | 0.00483 | 0.1763 |
| 10 | 0.33328 | 0.507166 |
| 11 | 0.096603 | 1.248594 |
| 12 | 0.062792 | 2.057643 |
| 13 | 0 | 2.767675 |
| 14 | 0 | 0.854936 |
| 15 | 0.096603 | 2.248434 |
| 16 | 0 | 2.057643 |
| 17 |  | 0.990181 |
| 18 |  | 1.666401 |
| 19 |  | 1.021577 |
| 20 |  | 0.016906 |
| 21 |  | 0.326035 |
| 22 |  | 0.268073 |
|  |  |  |
| mean | 0.08755 | 1.247 |
|  |  |  |
| Comparison | P-Value |  |
| Soma vs Exopher | 0.0011 |  |
|  |  |  |

**Numerical data for Figure 6B –** the volume of overlapping signal in 3-D projections between the hypodermal RAB-35 and ALMR-neuron derived exopher, comparing to the ALMR neuronal soma.

| sample | soma | exopher |
| --- | --- | --- |
| 1 | 0.024151 | 2.045568 |
| 2 | 0.316375 | 1.205122 |
| 3 | 0.195621 | 0.644825 |
| 4 | 0.219772 | 0.929804 |
| 5 | 0.473355 | 0.533731 |
| 6 | 0 | 1.922399 |
| 7 | 0.070037 | 0.007245 |
| 8 | 0.065207 | 3.670913 |
| 9 | 0.062792 | 5.042676 |
| 10 | 0.219772 | 6.668021 |
| 11 | 0.045886 | 0.417808 |
| 12 | 0 |  |
|  |  |  |
| mean | 0.1411 | 2.099 |
|  |  |  |
| Comparison | P-Value |  |
| Soma vs Exopher | 0.0047 |  |
|  |  |  |

**Numerical data for Figure 6C** **–** the volume of overlapping signal in 3-D projections between the hypodermal ARF-6 and ALMR-neuron derived exopher, comparing to the ALMR neuronal soma.

| sample | soma | exopher |
| --- | --- | --- |
| 1 | 0.016906 | 0.270488 |
| 2 | 0.270488 | 0.31879 |
| 3 | 0.079697 | 1.961041 |
| 4 | 0.301884 | 2.943976 |
| 5 | 0 | 1.036067 |
| 6 | 0.031396 | 0.246338 |
| 7 | 0.031396 | 0.396072 |
| 8 | 0.041056 | 3.296577 |
| 9 | 0 | 0.14732 |
| 10 | 0.002415 | 1.702628 |
| 11 | 0.002415 | 0.026566 |
| 12 | 0.007245 | 0.055547 |
| 13 | 0.057962 | 0.207696 |
| 14 | 0.082113 | 0.509581 |
| 15 | 0.00966 | 1.666401 |
| 16 | 0.173885 | 1.328291 |
| 17 | 0.002415 | 0.127999 |
| 18 | 0.007245 | 0.108678 |
| 19 | 0 | 0.425053 |
| 20 | 0 | 0.796975 |
| 21 | 0.062792 | 0.205281 |
| 22 | 0 | 0.188376 |
| 23 | 0.019321 | 0.070037 |
| 24 | 0.045886 | 0.135244 |
|  |  |  |
| mean | 0.05192 | 0.5926 |
|  |  |  |
| Comparison | P-Value |  |
| Soma vs Exopher | 0.0012 |  |
|  |  |  |

**Numerical data for Figure 6D** **–**exopher frequency in wild-type, *cnt-1(tm2313)*, *rab-35(b1034)* and *arf-6(tm1447)* mutants with mCherry expressed in the ALMR neurons.

| trial | wild-type | *cnt-1(tm2312)* | *rab-35(b1034)* | *arf-6(tm1447)* |
| --- | --- | --- | --- | --- |
| 1 | 4.1 | 21 | 15.9 | 0 |
| 2 | 3.8 | 13.75 | 15 | 1.9 |
| 3 | 5.2 | 12 | 11.1 | 0 |
| 4 | 7.9 | 12.98 | 9.2 | 1.7 |
| 5 | 1.3 | 12 | 13.3 | 1.9 |
|  |  |  |  |  |
| P-Value  WT vs mutant |  | 0.000296 | 0.001435 | 0.049215 |

**Numerical data for Figure 6E** **–**exopher frequency in wild-type and *arf-6(tm1447)* mutants with GFP expressed in the ALMR neurons.

| trial | wild-type | *arf-6(tm1447)* |
| --- | --- | --- |
| 1 | 8 | 0 |
| 2 | 6 | 2 |
| 3 | 8 | 0 |
| 4 | 11.5 | 4.8 |
|  |  |  |
| *p* value: |  | 0.003 |

| trial | wild-type | *cnt-1(tm2312)* | *rab-35(b1034)* | *arf-6(tm1447)* |
| --- | --- | --- | --- | --- |
| 1 | 4.1 | 21 | 15.9 | 0 |
| 2 | 3.8 | 13.75 | 15 | 1.9 |
| 3 | 5.2 | 12 | 11.1 | 0 |
| 4 | 7.9 | 12.98 | 9.2 | 1.7 |
| 5 | 1.3 | 12 | 13.3 | 1.9 |
|  |  |  |  |  |
| P-Value  WT vs mutant |  | 0.000296 | 0.001435 | 0.049215 |

| trial | wild-type | *cnt-1(tm2312)* | *rab-35(b1034)* | *arf-6(tm1447)* |
| --- | --- | --- | --- | --- |
| 1 | 4.1 | 21 | 15.9 | 0 |
| 2 | 3.8 | 13.75 | 15 | 1.9 |
| 3 | 5.2 | 12 | 11.1 | 0 |
| 4 | 7.9 | 12.98 | 9.2 | 1.7 |
| 5 | 1.3 | 12 | 13.3 | 1.9 |
|  |  |  |  |  |
| P-Value  WT vs mutant |  | 0.000296 | 0.001435 | 0.049215 |

**Numerical data for Figure 6F** **–**exopher frequency in wild-type and *arf-6(gf)* gain-of-funtion mutants with mCherry expressed in the ALMR neurons.

| trial | wild-type | *arf-6(gf)* |
| --- | --- | --- |
| 1 | 6 | 20 |
| 2 | 4 | 10 |
| 3 | 6 | 14 |
| 4 | 4 | 4 |
| 5 | 4 | 16 |
|  |  |  |
| *p* value: |  | 0.003 |

**Numerical data for Figure 6G** **–**exopher frequency in wild-type, *cnt-1* and *rab-35* single mutants and double mutants with *arf-6*.

| trial | wild-type | *rab-35(-)* | *rab-35(-); arf-6(-)* | *cnt-1(-)* | *cnt-1(-); arf-6(-)* |
| --- | --- | --- | --- | --- | --- |
| 1 | 2.3 | 15 | 6.8 | 9.1 | 3.4 |
| 2 | 4 | 15 | 11.5 | 9.3 | 1.1 |
| 3 | 6.5 | 14.3 | 8.8 | 14 | 3.8 |
| 4 | 3.2 | 7.9 | 1.9 | 8.2 | 2.9 |
| 5 | 4.8 | 11.5 | 2 | 12 | 3.3 |
|  |  |  |  |  |  |
| P-Value  WT vs mutant |  | 0.0010979 | 0.4098784 | 0.0110330 | 0.7972070 |
| P-Value  single mutant vs double mutant |  |  | 0.0195334 |  | 0.0015084 |

**Numerical data for Figure 6H-J–** Rescue experiments

**6H**- exopher frequency in *cnt-1* mutant and hypodermis-specific/neuron-specific expression of *cnt-1* in its cognate mutant

| trial | *cnt-1(-)* | *cnt-1(-);hyp cnt-1(+)* | *cnt-1(-);neuron cnt-1(+)* |
| --- | --- | --- | --- |
| 1 | 16.4 | 5.2 | 7.1 |
| 2 | 21.8 | 8.6 | 7.9 |
| 3 | 15.8 | 7.3 | 16.4 |
| 4 | 10 | 10.5 | 23.6 |
| 5 | 9.8 | 13.5 | 5.8 |
| 6 | 20 | 8.2 | 7.5 |
| 7 | 26.9 | 5.5 | 8.9 |
| 8 | 20.4 | 7.4 | 21.4 |
| 9 | 8.6 | 6.7 |  |
| 10 | 23.2 | 5.4 |  |
| 11 | 11.1 | 6.9 |  |
|  |  |  |  |
| P-Value  Compared to *cnt-1(-)* |  | 0.0002 | 0.1686 |

**6H**- starry night frequency in *cnt-1* mutant and hypodermis-specific/neuron-specific expression of *cnt-1* in its cognate mutant

| trial | *cnt-1(-)* | *cnt-1(-);hyp cnt-1(+)* | *cnt-1(-);neuron cnt-1(+)* |
| --- | --- | --- | --- |
| 1 | 0 | 5.2 | 0 |
| 2 | 0 | 10.3 | 0 |
| 3 | 3.5 | 7.3 | 1.8 |
| 4 | 3.3 | 5.3 | 5.5 |
| 5 | 1.96 | 3.8 | 3.8 |
| 6 | 2 | 4.1 | 0 |
| 7 | 3.8 | 21.8 | 1.8 |
| 8 | 3.7 | 0 | 0 |
| 9 | 3.4 | 6.7 |  |
| 10 | 3.6 | 7.1 |  |
| 11 | 3.7 | 6.9 |  |
|  |  |  |  |
| P-Value  Compared to *cnt-1(-)* |  | 0.0164 | 0.2239 |

**Numerical data for Figure 6H-J–** Rescue experiments

**6I**- exopher frequency in *rab-35* mutant and hypodermis-specific/neuron-specific expression of *rab-35* in its cognate mutant

| trial | rab-35(-) | rab-35(-);hyp rab-35(+) | rab-35(-);neuron rab-35(+) |
| --- | --- | --- | --- |
| 1 | 10.7 | 1.7 | 7.7 |
| 2 | 10.9 | 3.9 | 3.8 |
| 3 | 5.8 | 5.5 | 2.8 |
| 4 | 14.3 | 9.1 | 19.6 |
| 5 | 9.3 | 15.4 | 12.3 |
| 6 | 20.8 | 6.4 | 5.4 |
| 7 | 20.1 | 5.7 | 20 |
| 8 | 19.6 | 15 | 11.9 |
|  |  |  |  |
| P-Value  Compared to *rab-35(-)* |  | 0.0386 | 0.2791 |

**6I**- starry night frequency in *rab-35* mutant and hypodermis-specific/neuron-specific expression of *rab-35* in its cognate mutant

| trial | rab-35(-) | rab-35(-);hyp rab-35(+) | rab-35(-);neuron rab-35(+) |
| --- | --- | --- | --- |
| 1 | 0 | 3.4 | 1.9 |
| 2 | 0 | 5.9 | 3.8 |
| 3 | 3.8 | 3.6 | 0.9 |
| 4 | 3.6 | 9.1 | 1.8 |
| 5 | 0 | 5.1 | 1.8 |
| 6 | 1.9 | 8.5 | 0 |
| 7 | 4.2 | 7.5 | 0 |
| 8 | 2.2 | 25 | 6.8 |
|  |  |  |  |
| P-Value  Compared to *rab-35(-)* |  | 0.0224 | 0.8756 |

**Numerical data for Figure 6H-J–** Rescue experiments

**6J**- exopher frequency in wild-type, *arf-6* mutant and hypodermis-specific expression of *arf-6* in its cognate mutant

| trial | WT | arf-6(-) | arf-6(-);hyp arf-6(+) |
| --- | --- | --- | --- |
| 1 | 5.3 | 1.9 | 5.1 |
| 2 | 3.5 | 1.8 | 0 |
| 3 | 3.9 | 3.6 | 3.8 |
| 4 | 1.75 | 2 | 1.9 |
| 5 | 6 | 0 | 1 |
| 6 | 9.25 | 2.8 | 5.3 |
| 7 | 2.7 | 0 | 7.5 |
|  |  |  |  |
| P-Value  Compared to wild-type |  | 0.0191 | 0.4364 |

**6J**- starry night frequency in in wild-type, *arf-6* mutant and hypodermis-specific expression of *arf-6* in its cognate mutant

| trial | WT | arf-6(-) | arf-6(-);hyp arf-6(+) |
| --- | --- | --- | --- |
| 1 | 3.5 | 1.9 | 1.8 |
| 2 | 4.3 | 0 | 7.5 |
| 3 | 7 | 1.9 | 6.5 |
| 4 | 5.9 | 0 | 2 |
| 5 | 6 | 0 | 1.8 |
| 6 | 9.3 | 1 | 1.9 |
| 7 | 4.5 | 1.1 | 5.6 |
|  |  |  |  |
| P-Value  Compared to wild-type |  | 5.20167E-05 | 0.1411 |

**Numerical data for Figure 6K** **–**exopher and starry night frequency in *daf-2* mutant and the double mutant with *daf-2* and *arf-6*.

| trial | *daf-2(-)* exopher | *daf-2(-); arf-6(-) exopher* | *daf-2(-)*  starry night | *daf-2(-); arf-6(-) starry night* |
| --- | --- | --- | --- | --- |
| 1 | 47 | 10 | 19 | 10 |
| 2 | 60 | 15 | 16 | 15 |
| 3 | 64 | 15 | 22 | 21 |
| 4 | 47 | 20 | 26 | 10 |
| 5 | 55 | 6.2 | 22 | 11 |
| 6 | 42 | 12 | 23 | 1.7 |
| 7 | 60 | 14 | 15 | 5.4 |
|  |  |  |  |  |
| P-Value  Compared to *daf-2(-)* |  | 8.57036E-08 |  | 0.004221783 |

**Numerical data for Figure 6L** **–** attached exopher rate in *daf-2* mutant and the double mutant with *daf-2* and *arf-6*.

| trial | *daf-2(-)* | *daf-2(-); arf-6(-)* |
| --- | --- | --- |
| 1 | 0 | 17 |
| 2 | 2.9 | 37.5 |
| 3 | 0 | 25 |
| 4 | 0 | 25 |
| 5 | 2.7 | 0 |
| 6 | 8 | 14 |
| 7 | 3.6 | 12.5 |
|  |  |  |
| P-Value  Compared to *daf-2(-)* |  | 0.00423016 |
