# Supplementary material for: Large vesicle extrusions from C. elegans neurons are consumed and stimulated by glial-like phagocytosis activity of the neighboring cell
Source: eLife. 2023 Mar 2;12:e82227. doi: 10.7554/eLife.82227 (PMC10023159; doi:10.7554/eLife.82227)
Supplement: Figure 6—figure supplement 1—source data 1. [file elife-82227-fig6-figsupp1-data1.docx]

**Numerical data for Figure 6 - Figure supplement 1 –** exopher numbers at different days of adulthood in wild-type and *cnt-1(tm2313)* mutant

| day | wild-type | | | *cnt-1(tm2313)* | | |
| --- | --- | --- | --- | --- | --- | --- |
| 1 | 0 | 5.6 | 2 | 5.9 | 6 | 13.2 |
| 2 | 7.1 | 7.7 | 3.4 | 8.5 | 8.1 | 10.9 |
| 3 | 4 | 4.3 | 1.9 | 11.3 | 8 | 7.7 |
| 4 | 3.4 | 4.3 | 1.8 | 6.8 | 11.8 | 10.6 |
| 5 | 0 | 2.5 |  | 4.7 | 12.5 | 10.5 |

**Numerical data for Figure 6 - Figure supplement 1 –** starry night numbers at different days of adulthood in wild-type and *cnt-1(tm2313)* mutant

| day | wild-type | | | *cnt-1(tm2313)* | | |
| --- | --- | --- | --- | --- | --- | --- |
| 1 | 0 | 1.9 | 0 | 0 | 0 | 0 |
| 2 | 1.8 | 3.6 | 5.8 | 1.7 | 9.7 | 3.6 |
| 3 | 14 | 13 | 8.9 | 9.4 | 4 | 5.8 |
| 4 | 8.5 | 3.6 | 6.5 | 17 | 17.6 | 2.7 |
| 5 | 2 | 2.5 |  | 14.1 | 2.6 | 7.9 |
