# Supplementary material for: Large vesicle extrusions from C. elegans neurons are consumed and stimulated by glial-like phagocytosis activity of the neighboring cell
Source: eLife. 2023 Mar 2;12:e82227. doi: 10.7554/eLife.82227 (PMC10023159; doi:10.7554/eLife.82227)
Supplement: Figure 6—figure supplement 2—source data 1. [file elife-82227-fig6-figsupp2-data1.docx]

**Numerical data for Figure 6 - Figure supplement 2–** exopher frequency in wild-type, *cnt-1(tm2313)* and hypodermis-specific overexpression of *rab-35(+)* in *cnt-1(tm2313)* mutant

| trial | WT | *cnt-1(-)* | hyp *rab-35; cnt-1(-)* |
| --- | --- | --- | --- |
| 1 | 1.7 | 9.1 | 11 |
| 2 | 5.3 | 9.3 | 7.5 |
| 3 | 3.5 | 5.5 | 3.8 |
| 4 | 3.8 | 5.8 | 5.5 |
| 5 | 1.7 | 8.5 | 4.8 |
| 6 | 5.2 | 8.2 | 4.8 |
| 7 | 1.7 | 9.1 | 11 |
|  |  |  |  |
| P-Value  Compared to wild-type |  | 0.02641 |  |
| P-Value  Compared to *cnt-1(-)* |  |  | 0.3729 |

**Numerical data for Figure 6 - Figure supplement 2–** exopher frequency in wild-type, *rab-35(b1034)* and hypodermis-specific overexpression of *cnt-1(+)* in *rab-35(b1034)* mutant

| trial | WT | *rab-35(-)* | hyp *cnt-1*; *rab-35(-)* |
| --- | --- | --- | --- |
| 1 | 1.7 | 15 | 11.1 |
| 2 | 5.3 | 15 | 6.2 |
| 3 | 3.5 | 14.3 | 10.5 |
| 4 | 3.8 | 7.9 | 4.5 |
| 5 | 1.7 | 11.5 | 3.3 |
| 6 | 5.2 | 7.5 | 3.4 |
|  |  |  |  |
| P-Value  Compared to wild-type |  | 3.3E-05 |  |
| P-Value  Compared to *rab-35(-)* |  |  | 0.00063 |
