# Supplementary material for: Large vesicle extrusions from C. elegans neurons are consumed and stimulated by glial-like phagocytosis activity of the neighboring cell
Source: eLife. 2023 Mar 2;12:e82227. doi: 10.7554/eLife.82227 (PMC10023159; doi:10.7554/eLife.82227)
Supplement: Figure 6—figure supplement 3—source data 1. [file elife-82227-fig6-figsupp3-data1.docx]

**Numerical data for Figure 6 - Figure supplement 3 –**the total intensity of hypodermal ARF-6::mNeonGreen signal around the exopher periphery in wild-type and *cnt-1(tm2313)* mutant

| sample | wild-type | *cnt-1(tm2313)* |
| --- | --- | --- |
| 1 | 18874 | 276273 |
| 2 | 15790 | 222579 |
| 3 | 36118 | 199131 |
| 4 | 35849 | 112752 |
| 5 | 17095 | 34713 |
| 6 | 16123 | 144219 |
| 7 | 29548 | 78653 |
| 8 | 48632 | 180140 |
| 9 | 32039 | 212802 |
| 10 | 49486 | 258844 |
| 11 | 42215 | 22298 |
| 12 | 52972 | 47466 |
| 13 | 39963 | 62284 |
| 14 | 98575 | 62284 |
| 15 | 83340 | 78010 |
| 16 | 34779 | 67138 |
| 17 | 54610 | 232743 |
| 18 | 29045 | 703528 |
| 19 | 90793 |  |
| 20 | 62510 |  |
| 21 | 89372 |  |
| 22 | 372544 |  |
| 23 | 50430 |  |
| 24 | 75910 |  |
| 25 | 81863 |  |
| 26 | 153313 |  |
|  |  |  |
| mean | 65838 | 166437 |
|  |  |  |
| Comparison | P-Value |  |
| WT vs mutant | 0.0063 |  |
|  |  |  |
