# Supplementary material for: Large vesicle extrusions from C. elegans neurons are consumed and stimulated by glial-like phagocytosis activity of the neighboring cell
Source: eLife. 2023 Mar 2;12:e82227. doi: 10.7554/eLife.82227 (PMC10023159; doi:10.7554/eLife.82227)
Supplement: Figure 6—figure supplement 4—source data 1. [file elife-82227-fig6-figsupp4-data1.docx]

**Numerical data for Figure 6 - Figure supplement 4 –** *arf-6* mutants maintain early ALM budding

|  | *daf-2(e1370)* | *arf-6(tm1447);daf-2(e1370)* | CMH test |
| --- | --- | --- | --- |
| Bud events | 17 | 43 | P=0.0605 |
| Total observed neurons | 50 | 82 |  |
