# Supplementary material for: Large vesicle extrusions from C. elegans neurons are consumed and stimulated by glial-like phagocytosis activity of the neighboring cell
Source: eLife. 2023 Mar 2;12:e82227. doi: 10.7554/eLife.82227 (PMC10023159; doi:10.7554/eLife.82227)
Supplement: Figure 7—source data 1. [file elife-82227-fig7-data1.docx]

**Numerical data for Figure 7A** **–** exopher and starry night frequency in the hypodermal-specific RNAi for empty vector control (L4440) and *arf-6*

| trial | L4440_exopher | *arf-6_exopher* | L4440_starry night | *arf-6_starry night* |
| --- | --- | --- | --- | --- |
| 1 | 10 | 2.7 | 2.7 | 2 |
| 2 | 11.3 | 0 | 1.6 | 0 |
| 3 | 4.8 | 1.9 | 3.4 | 1 |
| 4 | 3.7 | 1.8 | 5.4 | 1 |
|  |  |  |  |  |
| P-Value  Compared to L4440 |  | 0.0116 |  | 0.0444 |

**Numerical data for Figure 7B** **–** exopher and starry night frequency in the hypodermal-specific RNAi for empty vector control (L4440) and *pkk-1*

| trial | L4440_exopher | *ppk-1_exopher* | L4440_starry night | *ppk-1_starry night* |
| --- | --- | --- | --- | --- |
| 1 | 10 | 8.5 | 2.7 | 1.7 |
| 2 | 1.9 | 8.3 | 8.3 | 0 |
| 3 | 4.8 | 6.1 | 3.4 | 1 |
| 4 | 3.7 | 4.7 | 5.4 | 1.9 |
|  |  |  |  |  |
| P-Value  Compared to L4440 |  | 0.3948 |  | 0.0286 |

**Numerical data for Figure 7C** **–** exopher and starry night frequency in the hypodermal-specific RNAi for empty vector control (L4440) and *pld-1*

| trial | L4440_exopher | *pld-1_exopher* | L4440_starry night | *pld-1_starry night* |
| --- | --- | --- | --- | --- |
| 1 | 10 | 12.3 | 2.7 | 6.3 |
| 2 | 11.3 | 12.5 | 1.6 | 5.8 |
| 3 | 1.9 | 2.1 | 8.3 | 0 |
| 4 | 3.6 | 5.8 | 3.4 | 2 |
| 5 | 8.9 | 2 | 3.3 | 7 |
|  |  |  |  |  |
| P-Value  Compared to L4440 |  | 0.9481 |  | 0.9820 |

**Numerical data for Figure 7D** **–** exopher and starry night frequency in the hypodermal-specific RNAi for empty vector control (L4440) and *sec-10*

| trial | L4440_exopher | *sec-10_exopher* | L4440_starry night | *sec-10_starry night* |
| --- | --- | --- | --- | --- |
| 1 | 10 | 3.7 | 3.3 | 0 |
| 2 | 3.6 | 0 | 5 | 3.6 |
| 3 | 3.2 | 3.6 | 6.5 | 0 |
| 4 | 6.7 | 1.7 | 7.3 | 3.8 |
| 5 | 3.6 | 0 | 2.7 | 1.9 |
| 6 | 8.9 | 3.8 | 5.4 | 4.9 |
|  |  |  |  |  |
| P-Value  Compared to L4440 |  | 0.0218 |  | 0.0378 |

**Numerical data for Figure 7E** **–** exopher and starry night frequency in the hypodermal-specific RNAi for empty vector control (L4440) and *rfip-1*

| trial | L4440_exopher | *rfip-1_exopher* | L4440_starry night | *rfip-1_starry night* |
| --- | --- | --- | --- | --- |
| 1 | 3.6 | 3.6 | 5 | 1.6 |
| 2 | 1.7 | 3.6 | 7.4 | 4.8 |
| 3 | 3.2 | 4.8 | 6.5 | 2 |
| 4 | 6.7 | 1.9 | 7.3 | 6.7 |
| 5 | 6.8 | 3.3 | 3.3 | 1.8 |
| 6 | 4.8 | 8.3 | 3.4 | 7.5 |
|  |  |  |  |  |
| P-Value  Compared to L4440 |  | 0.8624 |  | 0.3076 |

**Numerical data for Figure 7F** **–** exopher and starry night frequency in the hypodermal-specific RNAi for empty vector control (L4440) and *unc-16*

| trial | L4440_exopher | *unc-16_exopher* | L4440_starry night | *unc-16_starry night* |
| --- | --- | --- | --- | --- |
| 1 | 10 | 5.7 | 2.7 | 6.3 |
| 2 | 11.3 | 11.5 | 1.6 | 5.8 |
| 3 | 4.8 | 6.8 | 8.3 | 0 |
| 4 | 5.5 | 9.7 | 3.4 | 2 |
| 5 | 3.7 | 12.5 | 5.4 | 7 |
|  |  |  |  |  |
| P-Value  Compared to L4440 |  | 0.3071 |  | 0.9743 |

**Numerical data for Figure 7G–** attached exopher rate in the hypodermal-specific RNAi for empty vector control (L4440) and *ppk-1*

| trial | L4440 | *ppk-1* |
| --- | --- | --- |
| 1 | 25 | 100 |
| 2 | 14.3 | 50 |
| 3 | 33.3 | 50 |
| 4 | 25 | 71.4 |
| 5 | 25 | 80 |
| 6 | 50 | 100 |
| 7 | 66.6 | 60 |
|  |  |  |
| P-Value  Compared to *daf-2(-)* |  | 0.003142264 |
