# Supplementary material for: Large vesicle extrusions from C. elegans neurons are consumed and stimulated by glial-like phagocytosis activity of the neighboring cell
Source: eLife. 2023 Mar 2;12:e82227. doi: 10.7554/eLife.82227 (PMC10023159; doi:10.7554/eLife.82227)
Supplement: Figure 8—source data 1. [file elife-82227-fig8-data1.docx]

**Numerical data for Figure 8A** **–** exopher and starry night numbers in wild-type and *ced-1(e1735)* mutant

| trial | wild-type exopher | *ced-1(e1735) exopher* | wild-type starry night | *ced-1(e1735) starry night* |
| --- | --- | --- | --- | --- |
| 1 | 10 | 1.7 | 22 | 1.7 |
| 2 | 14 | 3.4 | 21 | 0 |
| 3 | 15 | 0 | 10 | 2 |
| 4 | 17 | 1.9 | 5 | 1.9 |
| 5 | 13 | 1.8 | 9 | 0 |
| 6 | 16 | 3.6 | 33 | 7.1 |
| 7 | 16 | 1.8 | 43 | 3.5 |
| P-Value  Compared to wild-type |  | 3.53971E-08 |  | 0.005148621 |

**Numerical data for Figure 8B** **–** exopher numbers (%) in wild-type, *ced-1(e1735)* mutant and the hypodermal rescue mutant

| trial | Wild type | *ced-1(e1735)* | *ced-1(e1735); hyp ced-1(+)* |
| --- | --- | --- | --- |
| 1 | 16 | 2 | 12 |
| 2 | 10 | 2 | 4 |
| 3 | 12 | 2 | 10 |
|  |  |  |  |
| P-Value  Compared to wild-type |  | 0.0009 | 0.35 |
| P-Value  Compared to ced-1(e1735) |  |  | 0.02 |

**Numerical data for Figure 8C –** the volume of overlapping signal in 3-D projections between the hypodermal CED-1 and ALMR-neuron derived exopher, comparing to the ALMR neuronal soma.

| sample | soma | exopher |
| --- | --- | --- |
| 1 | 0 | 5.610218 |
| 2 | 0 | 0.294639 |
| 3 | 0.00483 | 0.125584 |
| 4 | 0 | 0 |
| 5 | 0.055547 | 0.00483 |
| 6 | 0.229432 | 0.031396 |
| 7 | 0 | 0.055547 |
| 8 | 0.012203 | 4.861545 |
| 9 | 0 | 0.028981 |
| 10 | 0.097627 | 7.505106 |
| 11 | 0.781019 | 0.756612 |
| 12 | 0.103729 | 6.821714 |
| 13 | 0 | 8.170193 |
| 14 | 0.073221 | 4.795946 |
| 15 | 0.195255 | 0.305086 |
| 16 | 0.018305 | 6.162729 |
| 17 | 0 | 6.504425 |
| 18 |  | 4.295605 |
| 19 |  | 2.904415 |
| 20 |  | 4.2773 |
| 21 |  | 9.6224 |
| 22 |  | 4.356622 |
| 23 |  | 1.677971 |
| 24 |  | 9.79935 |
| 25 |  | 6.296967 |
| mean | 0.09242 | 3.811 |
|  |  |  |
| Comparison | P-Value |  |
| Soma vs Exopher | 3.87851E-05 |  |
|  |  |  |
|  |  |  |

**Numerical data for Figure 8D–** exopher frequency in wild-type, *ttr-52(tm2078)*, and *anoh-1(tm4762)* mutants.

| trial | WT | *ttr-52(tm2078)* | *anoh-1(tm4762)* |
| --- | --- | --- | --- |
| 1 | 11.3 | 5.9 | 4.2 |
| 2 | 14.3 | 3.5 | 2.2 |
| 3 | 15.7 | 4.6 | 3.1 |
| 4 | 12.5 | 5.8 | 1.7 |
|  |  |  |  |
| P-Value  Compared to wild-type |  | 0.005750317 | 0.000214919 |

**Numerical data for Figure 8D–** starry night frequency in in wild-type, *ttr-52(tm2078)*, and *anoh-1(tm4762)* mutants.

| trial | WT | ttr-52(tm2078) | anoh-1(tm4762) |
| --- | --- | --- | --- |
| 1 | 28.3 | 0 | 2.1 |
| 2 | 28.6 | 3.5 | 4.4 |
| 3 | 19.6 | 0 | 0 |
| 4 | 23.2 | 0 | 8.5 |
|  |  |  |  |
| P-Value  Compared to wild-type |  | 4.89045E-05 | 0.0003 |

**Numerical data for Figure 8E** **–** exopher and starry night numbers in wild-type and *ced-10(n3246)* mutant

| trial | wild-type exopher | *ced-10(n3246) exopher* | wild-type starry night | *ced-10(n3246) starry night* |
| --- | --- | --- | --- | --- |
| 1 | 10 | 29.3 | 16.7 | 4.9 |
| 2 | 8 | 35.6 | 8 | 2.2 |
| 3 | 9.3 | 40 | 14.8 | 2.5 |
| P-Value  Compared to wild-type |  | 1.58E-07 |  | 0.003545288 |

**Numerical data for Figure 8F** **–** exopher and starry night numbers in control and *ced-10* RNAi treated hypodermal specific RNAi strain

| trial | control exopher | *ced-10 RNAi exopher* | control starry night | *ced-10 RNAi starry night* |
| --- | --- | --- | --- | --- |
| 1 | 6.7 | 19.4 | 8.3 | 0 |
| 2 | 6.8 | 21.1 | 3.4 | 0 |
| 3 | 3.6 | 10.4 | 1.9 | 0 |
| 4 | 1.9 | 11.7 | 5.5 | 0 |
| 5 | 8.9 | 10.3 | 3.7 | 0 |
| P-Value  Compared to control |  | 0.0095 |  | 0.0032 |

**Numerical data for Figure 8G –** the volume of overlapping signal in 3-D projections between the hypodermal CED-10 and ALMR-neuron derived exopher, comparing to the ALMR neuronal soma.

| sample | soma | exopher |
| --- | --- | --- |
| 1 | 0.569958 | 0.4806 |
| 2 | 0.202866 | 0.1763 |
| 3 | 0.144904 | 0.007245 |
| 4 | 0.654485 | 2.453716 |
| 5 | 0 | 1.449045 |
| 6 | 0 | 0.036226 |
| 7 | 0.002415 | 1.205122 |
| 8 | 0.012075 | 1.026407 |
| 9 | 0 | 0.00966 |
| 10 | 0.055547 | 0.002415 |
| 11 | 0.304299 | 0.00966 |
| 12 | 0 | 0.393657 |
| 13 | 0.012075 | 0.342941 |
| 14 | 0 | 0 |
| 15 | 0.055547 | 0.01449 |
| 16 | 0.304299 | 0.212527 |
| 17 | 0 | 1.625345 |
| 18 | 0.038641 | 1.700212 |
| 19 | 0 | 0.968445 |
| 20 | 0.002415 | 2.668657 |
| 21 | 0 | 2.661412 |
| 22 | 0 | 0.722107 |
| 23 | 0.305086 | 0.113509 |
| 24 | 0.530849 | 0.198036 |
| 25 | 0.079322 | 0.487845 |
| 26 | 0.848138 | 0.086943 |
| 27 | 0 | 0.763164 |
| 28 | 0.122034 | 0.681051 |
|  |  |  |
| mean | 0.1516 | 0.6049 |
|  |  |  |
| Comparison | P-Value |  |
| Soma vs Exopher | 0.0059 |  |
|  |  |  |
|  |  |  |
