# Supplementary material for: Large vesicle extrusions from C. elegans neurons are consumed and stimulated by glial-like phagocytosis activity of the neighboring cell
Source: eLife. 2023 Mar 2;12:e82227. doi: 10.7554/eLife.82227 (PMC10023159; doi:10.7554/eLife.82227)
Supplement: Figure 8—figure supplement 2—source data 1. [file elife-82227-fig8-figsupp2-data1.docx]

**Numerical data for Figure 8 - Figure supplement 2 –**the touch sensitivity of arf-6 and ced-1 mutant

|  | AD5 | | AD10 | |
| --- | --- | --- | --- | --- |
| trial | WT | *arf-6* | WT | *arf-6* |
| 1 | 96 | 86 | 66.66 | 50 |
| 2 | 83.87 | 86.6 | 61.53 | 37.5 |
| 3 | 96.7 | 80 | 58.33 | 50 |
| 4 | 100 | 86.666 |  |  |
|  |  |  |  |  |
| *p* value as compared to the WT |  | 0.0532 |  | 0.0276 |

|  | AD5 | | AD10 | |
| --- | --- | --- | --- | --- |
| trial | WT | *ced-1* | WT | *ced-1* |
| 1 | 93.333 | 90 | 85.18 | 27.27 |
| 2 | 100 | 83.333 | 66.66 | 33.33 |
| 3 | 83.87 | 90 | 61.53 | 54.54 |
|  |  |  |  |  |
| *p* value as compared to the WT |  | 0.4226 |  | 0.0403 |
